# Comprehensive pharmacokinetic/pharmacodynamic analysis of commercially available premixed daptomycin preparations against *Staphylococcus aureus* and *Enterococcus* spp. bloodstream infections using Monte Carlo simulation in adult patients

**DOI:** 10.1093/jacamr/dlaf253

**Published:** 2026-01-09

**Authors:** Emily Jaime, Arsheena Yassin, Raj H Patel, Tanaya Bhowmick, Andras Farkas

**Affiliations:** Department of Pharmacy, Cooperman Barnabas Medical Center, 94 Old Short Hills Road, Livingston, NJ 07039, USA; Department of Pharmacy, Robert Wood Johnson University Hospital, 1 Robert Wood Johnson Place, New Brunswick, NJ 08901, USA; Department of Medicine, Cooperman Barnabas Medical Center, 94 Old Short Hills Road, Livingston, NJ 07039, USA; Department of Medicine, Rutgers Robert Wood Johnson Medical School, 125 Paterson Street, New Brunswick, NJ 08901, USA; Department of Pharmacy, Cooperman Barnabas Medical Center, 94 Old Short Hills Road, Livingston, NJ 07039, USA; Optimum Dosing Strategies, 63 Reeve Ave, Bloomingdale, NJ 07403, USA

## Abstract

**Objectives:**

The current approach to dosing daptomycin is primarily weight-based, requiring time-consuming sterile product preparation. Commercially available, premixed formulations have the potential to streamline workflow.

**Materials and methods:**

We conducted Monte Carlo simulations using pharmacokinetic (PK) models of daptomycin. PTA for the treatment of staphylococcal and enterococcal bloodstream infections for adjusted body weight (ABW) categories of 40–100 kg, creatinine clearances (CrCl) of 10–120 mL/min and for renal replacement therapy (RRT) were investigated. A 24-h area under the curve to minimum inhibitory concentration ratio (AUC_24h_/MIC) of  ≥ 666 and a free (*f*) AUC_24h_/MIC of  > 27.43 were used as the threshold for efficacy for staphylococcal and enterococcal infections, respectively. A PTA of  > 51.6% for achieving a trough level ≥ 24.3 mg/L in a 90 kg patient with a CrCl of 40 mL/min was used as the cut-off for toxicity. Resulting PD indices of the premixed formulations were compared with those achieved by the 6, 8 and 10 mg/kg dosing regimens.

**Results:**

The doses provided by the premixed bags demonstrated increased likelihood of target attainment over the weight-based approaches. Analysis for the toxicity index also showed comparable risk of exposure, although PTAs of patients with ABW of 80 kg or more and with the CrCl of 10 and 40 mL/min are likely to surpass the toxicity threshold when the 1000 mg bag is administered.

**Conclusions:**

Based on the results, the commercially available fixed-dose premixed formulations of daptomycin can be integrated into day-to-day clinical practice with the expected benefit of improved efficacy target attainment.

## Introduction

Daptomycin is a cyclic lipopeptide antibiotic that exerts rapid bactericidal activity against a wide range of Gram-positive bacteria, including MRSA and VRE, via calcium-dependent insertion into the bacterial membrane.^1^ Daptomycin is approved for the treatment of complicated skin and soft tissue infections (cSSTIs), right-sided infective endocarditis (RIE) caused by *Staphylococcus aureus* and bacteraemia associated with cSSTIs or RIE.^2^ Daptomycin is a hydrophilic drug with high protein binding (90%–93%) and is mainly eliminated by the kidneys (78%).^3^ Daptomycin’s PK is generally linear at doses up to at least 12 mg/kg/day. In patients with stage 4 chronic kidney disease (CKD) (CrCl < 30 mL/min) and in subjects undergoing haemodialysis or peritoneal dialysis, a dosage adjustment of daptomycin is recommended.^2^ Daptomycin exhibits concentration-dependent bactericidal activity and its AUC_24h_/MIC is the most relevant PK/PD index of efficacy.^4^ According to available studies, daptomycin AUC_24h_/MIC ≥ 666 and *f*AUC_24h_/MIC > 27.43 can be used as targets for therapeutic success against *S. aureus* and enterococcal infections, respectively, while the minimum concentration (C_min_) can be used to monitor the muscle toxicity with a target value < 24.3 mg/L.^5–7^

Premixed daptomycin infusions are commercially available and offer several advantages over traditional lyophilized formulations that require reconstitution.^8^ The premixed solutions are prepared by the manufacturer to precise concentrations, thereby minimizing the potential for dosing errors associated with manual preparation. Their availability also eliminates the need for pharmacy staff to reconstitute and dilute the medication, saving valuable time and resources. These preparations extend shelf life and permit more flexible storage requirements that support better inventory management and reduce the likelihood of medication waste.^8^ Premixed solutions also reduce the chances of medication administration errors; barcoding allows for bedside scanning to ensure the correct medication is administered to the right patient. A current limitation in published literature is the lack of available studies evaluating the PD target attainment of the premixed products in varied patient populations (e.g. renal replacement modalities), which hinders the widespread adoption of these preparations in clinical practice.

Monte Carlo simulation (MCS) is a valuable tool for determining optimal dosage regimens and assisting in the selection for appropriate empirical antibiotic therapies. It is able to link PD data with the PK profile to predict the probability of a certain therapeutic outcome, thereby improving the likelihood of antimicrobial dosing regimen effectiveness.^9^ The Monte Carlo method has shown great promise in drug development, especially in designing phase II/III clinical trials of antimicrobial agents, and it is versatile enough to assess the impact of dosage changes by integrating it with population PK modelling. This frequently leads to identifying optimal and more reliable alternative dosing strategies with antibacterial drugs (i.e. prolonged infusion of beta-lactams) to achieve a predefined therapeutic target.

In this study, we assessed the PTA of various commercially available premixed daptomycin dosage regimens relative to predefined efficacy and safety indices for bloodstream infections caused by *S. aureus* and *Enterococcus* spp. using MCS in adult patients across all levels of renal function, including those on RRT.

## Materials and methods

### Pharmacokinetic models and Monte Carlo simulation

Two previously published population PK models of daptomycin were used in this *in silico* study. The model by Xu *et al*. was originally developed by merging and analysing data from patients on continuous veno-venous haemodialysis (CVVHD) and continuous veno-venous haemodiafiltration (CVVHDF) with a PK database of a base model for daptomycin collected from patients with various levels of renal function.^10^ For intermittent haemodialysis (IHD), we used the model by Patel *et al*. that was derived from patients on thrice-weekly 3.5-h IHD regimens.^11^ MCS for 1000 subjects were performed for the standard weight-based (6 mg/kg, 8 mg/kg and 10 mg/kg) doses and the commercially available premixed bag regimens (available in 350, 500, 700  and 1000 mg strengths) at 10 kg ABW (40–100 kg) and 10 mL/min CrCl (10–120 mL/min) increments.^12^ We elected to use ABW as the basis of dose selection in this work (with adjustment factor of 0.4) to aid in achieving comparable systemic exposure that is likely to yield similar clinical outcomes across BMI categories of obese and non-obese patients.^12,13^

A continuous dialysis schedule was assumed for CVVHD(F), while for IHD, the standard thrice- weekly approach was utilized, with the weight-based dosing simulated at 6 mg/kg for the 48-h and 9 mg/kg for the 72-h dialysis intervals. In the case of IHD, the PTA achieved after the intra-dialytic dosing intervals of 48 h and 72 h [i.e. the time from hours 120 to 144 (IHD 0–24) and hours 144 to 168 (IHD 24–48)] was used in the comparisons. Dialysis clearance was treated as a piece-wise input function: turned on during dialysis and turned off at the end of dialysis. Readers are referred to the original studies for complete technical details of the RRT protocols. Total and free concentrations were simulated where the protein binding of daptomycin was assumed to be 91%.^14^ The AUC was determined using the trapezoidal rule and at 0.6-min intervals.

Standard two-compartment structural models with first-order elimination were used during simulations as available in the framework provided by the Individually Designed Optimum Dosing Strategies^TM^ 6.0 (ID-ODS^TM^; https://www.optimum-dosing-strategies.org/) application. ID-ODS^TM^ 6.0 is a simulation tool that supports both probabilistic forecasting and Bayesian adaptive feedback powered by the R software (version 4.1.3; Institute for Statistics and Mathematics; http://www.r-project.org/) with an extensive model library built from published population PK models.^15–17^ The published probability distributions of the parameter values from the selected two models were used to generate unbound (total concentration × 0.09) and total concentration-time curves for each dosing regimen in plasma.

### Pharmacodynamic targets

The predefined PD target for efficacy was the daptomycin AUC_24h_/MIC ≥ 666 for MRSA and *f*AUC_24h_/MIC *>* 27.43 for VRE.^5,6^ These indices and their respective magnitudes are unique to each of these pathogens and thought to be required for optimal bacterial kill in serious infections. To establish the AUC for every-other-day dosing when not on RRT, we calculated an average value for 24 h derived from the 48-h simulated interval. These indices were calculated for each weight-based regimen with focus on quantifying the PTA at the MIC90 values of 0.5 mg/L for MRSA and 2 mg/L for VRE in North America in 2023.^5,6,18^ To establish the value of interest for the toxicity index, we used the Xu *et al*.’s model to simulate the probability of C_min_ ≥ 24.3 mg/L in plasma at an ABW of 90 kg and CrCl of 40 mL/min for the 10 mg/kg dose.^10^ The associated probability value of 51.7% for reaching the surrogate marker of toxicity thought to be associated with increased risk of CPK elevations is then used as the cut-off, a value that is generally accepted in clinical practice, even when using daptomycin in moderate renal impairment.^19–22^

In order to simulate a likely worst-case scenario and acknowledge that the PD index related to efficacy is mainly driven by drug clearance, the values for the following categorical covariates were set identical during simulations using the Xu *et al*.’s model: gender to male and indication to RIE. Aiming to characterize the applicability of our simulation results to clinical practice, the evaluation focused on comparing PTAs of achieving safety and efficacy endpoints of weight-based dosing regimens with those of their counterparts, the weight-based doses rounded up to the nearest available commercial bag size, yielding the per cent change in PTAs from the use of premixed preparations instead. Additionally, full details of the PTA of each dosing regimen for the MICs of 0.06125  to 256 mg/L are also presented in the supplement (Tables S1 and S2, available as Supplementary data at *JAC-AMR* Online).

## Results

### Staphylococcus

**Figure 1.**
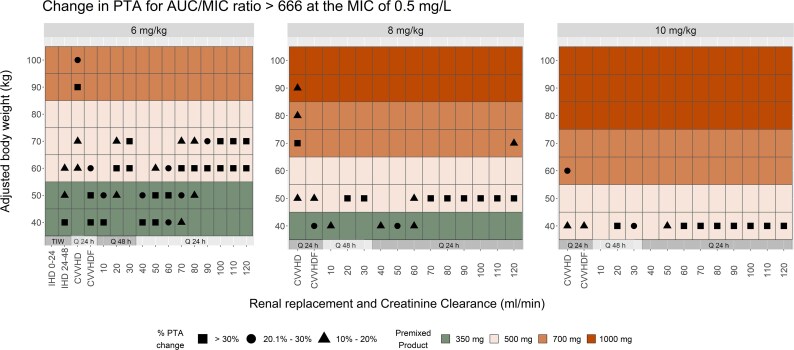
The shapes inside the tiles represent the per cent change in PTAs when the premixed bags are substituted for the weight-based regimens (only changes ≥ 10% are shown, for all other ABW and CrCl categories the PTA increase is 0 to  < 10%). As an example, patients with ABW of 70 kg and CrCl of 120 mL/min would benefit from an increase of  > 30% PTA when the 6 mg/kg (420 mg) weight-based dose is substituted to the 500 mg premixed bag (closed square). On the other hand, an increase of 10% to 20% PTA is expected when the 8 mg/kg (560 mg) weight-based dose is substituted to the 700 mg premixed bag (closed triangle). Finally, no change in PTA is expected for the 10 mg/kg weight-based dose, as the commercial bags size would be its equivalent (no shape inside the tile).

Output of the PTA analysis for the treatment of staphylococcal bloodstream infections are shown in Figure 1. The PTA for the premixed dosing regimens compared to the weight-based dosing approaches showed similarly robust outcomes across the ABW and renal function categories. An increase of at least 10% for 23.7% and an increase of at least 30% for 12.3% of all regimens evaluated was observed when investigating the likelihood of reaching the target AUC_24h_/MIC ≥ 666 at the MIC of 0.5 mg/L. The most notable target attainment gains were observed at the 60 kg ABW for the 6 mg/kg, 50 kg ABW for the 8 mg/kg and at the 40 kg ABW for the 10 mg/kg dosing approaches. PTAs of the premixed preparations for these ABW categories exceeded those of the weight-based regimens by 30% or more in 50%, 57.1% and 57.1% of renal function categories, respectively.

### Enterococcus

**Figure 2.**
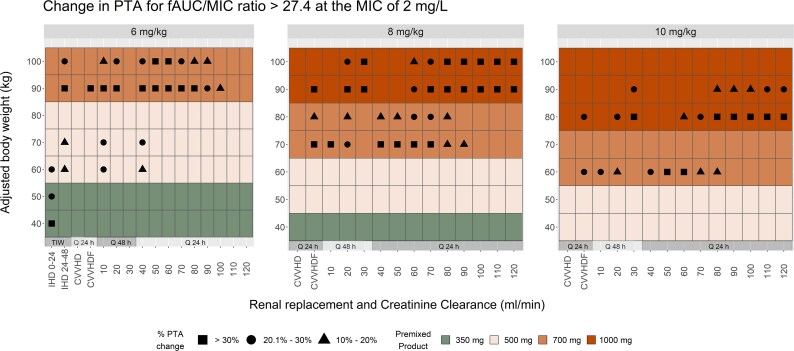
Same interpretation rules apply for this figure as for Figure 1.

Simulation results for the treatment of enterococcal bloodstream infection are even more compelling as they show an increase of at least 10% for 27.3% and an increase of at least 30% for 13% of all regimens evaluated when assessing the likelihood of reaching the target *f*AUC_24h_/MIC > 27.43 at the MIC of 2 mg/L (Figure 2.). Simulated subjects weighing 90 kg with 6 mg/kg, 90 kg with 8 mg/kg and 80 kg with 10 mg/kg dosing regimens are most likely to benefit from the increase in PTAs when substituting the total daily dose rounded up to the nearest available commercial preparation. The PTAs of the premixed preparations are expected to be higher than those of the weight-based regimens by 30% or more for these ABW categories in 50.0%, 64.2% and 42.8% of renal function tiers, respectively. Specific to RRT modalities, a slightly more modest benefit was observed that showed less than a 10% increase in PTAs for 83.9% of all regimens evaluated at the enterococcal efficacy targets. The PTAs of all other weight and renal function combinations are expected to achieve an increase within 10% or lower, when substituted with the premixed preparations.

### Safety

**Figure 3.**
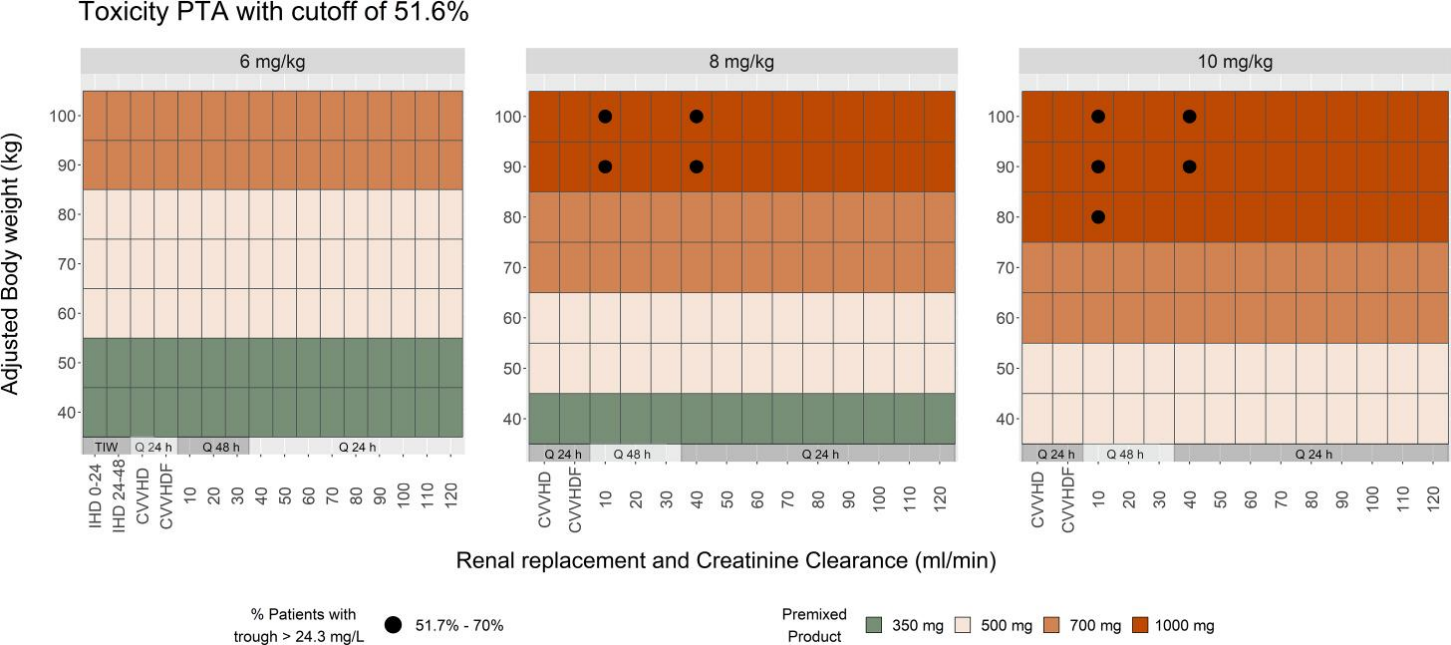
The shapes inside the tiles represent the per cent PTAs when the premixed bags are substituted for the weight-based regimens (only PTAs ≥ the cut-off of 51.7% are shown, for all other ABW and CrCl categories the PTA is  < 51.7%). As an example, patients with ABW of 80 kg and CrCl of 10 mL/min would be exposed to a risk level between 51.7% and 70% when the 10 mg/kg (800 mg) weight-based dose is substituted to the 1000 mg premixed bag (closed circle), a risk level higher than the predefined cut-off value. On the other hand, the risk is expected to be lower than the cut-off when the 8 mg/kg (640 mg) weight-based dose is substituted to the 700 mg premixed bag or when the 6 mg/kg (480 mg) weight-based dose is substituted to the 500 mg premixed bag (no shape inside the tile).

The proportions of dosing regimens predicted to have the PTA for the C_min_ total concentrations greater than the breakpoint value considered (i.e. PTA of C_min_ ≥ 24.3 mg/L of 51.7% based on a 90 kg patient receiving 10 mg/kg dose with a CrCl of 40 mL/min) were 0%, 4.1% and 5.1% for the commercially available preparations when substituted instead of the 6 , 8  and 10 mg/kg weight-based doses, respectively (Figure 3, solid black circles). After treatment with the high-dose 1000 mg premixed preparation, patients with CrCl of 10 and 40 mL/min and an ABW of 80 kg or more are likely to surpass the toxicity cut-off probability value indicated by the PTA range of 57.4% to 68.8% (Table S1).

## Discussion

In the present study, we used MCS analysis to investigate the ability of the commercially available premixed daptomycin preparations to achieve the PK/PD target associated with efficacy and safety indices when treating staphylococcal and enterococcal bloodstream infections. Based on these results, we developed a framework for interchangeability of the weight-based 6, 8  and 10 mg/kg/day dosing regimens commonly used in clinical practice based on ABW. Distinctive to this study, we conducted our analysis at a level of granularity uniquely suited for empiric dose individualization as we capture the majority of ABW and renal function categories encountered in clinical practice, including those treated with RRT. Overall, and as an attribute of our study design of doses rounded up to the nearest commercially available bag size, no losses in PTAs are expected when substituting the premixed preparations for the weight-based dosing regimens. Conversely, the use of higher doses offered by the commercial preparations substantially increased the PTA, which advocates for their relative advantage over the traditional weight-based dosing approaches and when the efficacy endpoints are considered. Likewise, the results of our simulation analysis confer a comparable safety profile, as more than 97% of all dosing strategies evaluated resulted in PTAs that are equal to or lower than our predefined probability cut-off value of 51.7%.

Optimal daptomycin exposure is best described by AUC_24h_/MIC. The target threshold of AUC_24h_/MIC ≥ 666 is associated with improved clinical outcomes and microbiological eradication of *S. aureus*, while *Enterococcus* spp. bloodstream infections necessitate a threshold of *f*AUC_24h_/MIC *>* 27.43.^5,6,23^ Standard dosing of daptomycin at 6 mg/kg/day has been shown to be sufficient for achieving PTA > 90% against susceptible *S. aureus* strains with MICs ≤ 0.5 mg/L, and our study confirms these findings. However, standard doses for isolates with higher MICs (e.g. 1–2 mg/L) fall short of attaining the AUC_24h_/MIC target in most simulated scenarios, and only doses of 8 mg/kg/day or higher are expected to reasonably overcome that.^24,25^

Although weight-based dosing of daptomycin is generally recommended, it is important to note that daptomycin clearance does not scale proportionally with weight, and as a consequence nor does the AUC_24h_.^26^ In the study by Falcone *et al*. evaluating data derived from critically ill patients, the higher doses based on body weight in heavier patients increased the PTA for efficacy but also increased the likelihood of toxicity as compared with weight-based regimens for the average body weight patients. Contrarily, patients weighing < 75 kg were at risk of sub-therapeutic exposure with weight-based dosing due to lower absolute doses, especially in the presence of augmented renal clearance.^5^ According to their findings, the use of an empiric fixed dose of 750 mg is predicted to achieve a comparable efficacy PTA with a lower chance of toxicity as compared with the use of 10 mg/kg in this population of patients. Since body weight is not a significant predictor for daptomycin clearance, there have been additional interests in fixed versus weight-based dosing of daptomycin to optimize the chance of achieving both staphylococcal and enterococcal efficacy targets. In a recent publication by Olney *et al*., simulations were performed to evaluate the probability of achieving AUC_0–24h_/MIC ≥ 666 with fixed and weight-based doses.^27^ This study included 31 patients with a median age of 50 years and weight of 74 kg (IQR 54, 156). Fixed-dose daptomycin (750 mg) resulted in similar exposure across weights with a median (IQR) AUC_0–24h_ of 819 mg·h/L (499, 1501) in patients weighing ≤ 74 kg and 749 mg·h/L (606, 1265) in patients weighing > 74 kg. This is similar to exposures reported previously from patients receiving weight-based doses of daptomycin 8–12 mg/kg/day. They also identified that fixed dosing increased the PTA for the *S. aureus* efficacy target in those with higher CrCl. In yet another paper, Butterfield–Cowper evaluated weight-based versus fixed dosing of daptomycin based on PK-PD targets in enterococcal bacteraemia. PTA was assessed for 8–12 mg/kg/day and various fixed doses (500 , 750, 1000 and 1500 mg daily) using three published PK models derived from distinct populations of patients.^28^ Overall, higher fixed doses (750–1000 mg) led to increased exposures compared with the standard weight-based doses of 8–12 mg/kg/day. These results are also consistent with our study showing premixed daptomycin bags were able to improve efficacy PTA versus the weight-based regimens.

Even though high-dose daptomycin has shown superior bactericidal activity and improved clinical outcomes in the treatment of severe Gram-positive infections, its safety requires careful evaluation.^7^ Renal function plays a pivotal role in daptomycin clearance and, by extension, trough elevation. In patients with impaired renal function, C_min_ levels rise disproportionately compared with those with normal clearance, thereby likely increasing the risk of toxicity. In a study by Bhavnani *et al*., a significant relationship was found between daptomycin C_min_ levels ≥ 24.3 mg/mL and creatine phosphokinase (CPK) elevations, particularly in patients receiving > 8 mg/kg/day.^7^ Close to a decade later in a small PK study, Yamada *et al.* used logistic regression analysis to examine the breakpoint of CPK elevation and observed that the risk was higher when C_min_ > 19.5 mg/L.^29^ Despite these concerns, available clinical evidence suggests that daptomycin, even at high doses, can be administered safely in patients with and without renal impairment, provided that dose adjustments and enhanced monitoring strategies are employed when needed. In the study by Vlashyn *et al*. analysing data from a cohort of 50 renally impaired patients, 30 of which were on IHD primarily for the treatment of Gram-positive bacteraemia, no symptomatic CPK elevations and only one asymptomatic elevation occurred with high-dose daptomycin (median dose of > 8 mg/kg).^30^ Furthermore, the EU-CORE registry, which included over 6000 patients and a substantial proportion with moderate-to-severe renal dysfunction, found that high-dose daptomycin (> 6 mg/kg/day) was not associated with a disproportionate increase in serious adverse events, even when used in renally impaired individuals.^31^ Importantly, the rates of CPK elevation and treatment discontinuation due to adverse events in the subset of patients with renal impairment were similar to those observed in individuals with preserved renal function, especially when dosing intervals were appropriately extended. Simulations in our study also corroborate the previously established standard to dose adjust in severe renal impairment, as after treatment with the high-dose 1000 mg premixed preparation, a fraction of the patients with CrCl of 40 mL/min and an ABW of 80 kg or more are likely to surpass the toxicity cut-off probability threshold. Adjusting to a dosing interval of 48 h will mitigate this risk at CrCl of 20 and 30 mL/min, but further accumulation of trough levels is expected even at the extended interval when managing dosing for a patient with CrCl 10 mL/min.

Besides the potential clinical benefits, utilizing these premixed preparations may also offer operational advantages that may be of interest for healthcare administrators. Schmidt *et al*. conducted a retrospective study involving patients receiving daptomycin therapy and evaluated clinical and pharmacoeconomic outcomes pre- and post-implementation of a standardized dosing protocol. The standardized dosing approach produced a reduction of 21 500 mg of drug waste over 4 months, which corresponded to a cost savings of $13 845 in a quarter, while maintaining infection cure rates.^32^ Moreover, safety outcomes remained stable, with no observed increase in adverse events, suggesting that standardized doses remained within safe therapeutic ranges. This is critical in ensuring that interventions designed to improve efficiency do not inadvertently compromise patient safety. Their findings illustrate that standardized daptomycin dosing protocols offer a robust, reproducible and effective method to reduce waste, improve therapeutic consistency and support broader institutional goals related to antimicrobial stewardship and cost containment.

The primary limitation of this work, we believe, is the use of *in silico* routines to delineate daptomycin exposure based on published PK models and PD targets, which could lead to accepting assumptions that may not entirely be suitable to all target populations. Such simulations cannot fully replicate the biological variability, physiological complexity and multi-factorial influences that characterize real-world clinical settings. As a result, the predictive validity of the computational outcomes remains provisional, and further verification through studies incorporating clinical outcome data is necessary to establish translational accuracy and clinical relevance. Nevertheless, it is reasonable to think that the results are accurate and applicable to a wide range of clinical scenarios encountered in hospitalized patients; the primary model used for this analysis is based on validated published work derived from a diverse population, including the critically ill, those with renal impairment, and on RRT.^10^ It is also important to note that our analysis focused exclusively on PK/PD metrics (e.g. AUC_24h_/MIC, C_min_), without incorporating clinical outcome data. While these PK/PD targets are useful surrogates, they may not perfectly correlate with actual clinical success or toxicity, especially in heterogeneous populations or when treating diseases other than bloodstream infections.^33^ Consider, for example, the exclusion of female gender-specific simulations and subsequent evaluation of changes in PTAs, notably when analysing the toxicity target owing to the female gender-specific decrease in daptomycin clearance. In this work, we chose to emphasize exposure parameters associated with efficacy and thus selected the male gender-based calculations with the higher clearance, as the evidence supporting daptomycin’s toxicity indices is comparatively limited. The bulk of evidence indicates that higher trough C_min_ levels of daptomycin—particularly at or above ∼24.3 mg/L—are associated with a markedly increased probability of CPK elevation. The specific threshold we quoted is derived from very limited patient numbers and exposure modelling, thus leading to important limitations that suggest it cannot be considered an absolute. Also, CPK elevation is a sensitive marker of inflammation and often secondary to other comorbidities the patient may be experiencing. Besides, the real-world clinical data with high-dose daptomycin thus far have not registered significantly higher rates of elevated CPK concentrations or toxicity in female gender patients when given the same weight-based dosing regimens as male patients. Further prospective studies measuring actual trough levels and linking to clinically significant outcomes (myopathy, rhabdomyolysis, discontinuation) may clarify the safe upper exposure limit for daptomycin. At last, and as it relates to our study’s limitations, real-world variables such as missed doses, drug interactions, comorbidities or variability in infusion times were also not accounted for. These factors can significantly influence drug exposure and limit the applicability of the simulated regimens.^34^

## Conclusions

In summary, a population PK model-based simulation approach was used in this work to assess risk versus benefit profile of using commercially available premixed preparations instead of weight-based dosing regimens of daptomycin for the treatment of staphylococcal and enterococcal bloodstream infections. The premixed preparations provide equivalent and at times, improved PD target attainment in plasma when treating *S. aureus* at the MIC values of 0.5 mg/L or less and when treating enterococcus at the MIC values of  ≤ 2 mg/L. From the toxicity point of view, a slight increase in the chances of surpassing trough levels thought to be associated with daptomycin’s adverse effects is likely to be expected in a small, specific subset of patients. In light of the results of this study and the abundance of existing clinical evidence on the safety and efficacy profile of daptomycin in a wide range of weight-based dosing regimens, implementation of a substitution protocol with the premixed preparation with low safety risk may be considered.

## Supplementary Material

dlaf253_Supplementary_Data

## Supplementary data

Tables S1 and S2 are available as Supplementary data at *JAC-AMR* Online.
